# Unifying ecology and macroevolution with individual-based theory

**DOI:** 10.1111/ele.12430

**Published:** 2015-03-27

**Authors:** James Rosindell, Luke J Harmon, Rampal S Etienne

**Affiliations:** 1 Department of Life Sciences, Imperial College LondonSilwood Park campus, Buckhurst Road, Ascot, SL5 7PY, UK; 2 Department of Biological Sciences and Institute for Bioinformatics and Evolutionary Studies (IBEST), University of IdahoMoscow, ID, USA; 3 Community and Conservation Ecology, Centre for Ecological and Evolutionary Studies, University of GroningenBox 11103, 9700 CC, Groningen, The Netherlands

**Keywords:** Ecology, fitness, individual-based model, lineages-through-time, macroevolution, neutral, phylogeny, selection, species abundance, theory

## Abstract

A contemporary goal in both ecology and evolutionary biology is to develop theory that transcends the boundary between the two disciplines, to understand phenomena that cannot be explained by either field in isolation. This is challenging because macroevolution typically uses lineage-based models, whereas ecology often focuses on individual organisms. Here, we develop a new parsimonious individual-based theory by adding mild selection to the neutral theory of biodiversity. We show that this model generates realistic phylogenies showing a slowdown in diversification and also improves on the ecological predictions of neutral theory by explaining the occurrence of very common species. Moreover, we find the distribution of individual fitness changes over time, with average fitness increasing at a pace that depends positively on community size. Consequently, large communities tend to produce fitter species than smaller communities. These findings have broad implications beyond biodiversity theory, potentially impacting, for example, invasion biology and paleontology.

## Introduction

Revealing the fundamental mechanisms behind the evolution of biodiversity and its assembly into ecological communities is a challenging task. It is increasingly recognised that the ecological and evolutionary aspects of biodiversity are intertwined (Schoener 2011). Consequently, a generalised theory that encompasses both ecology and evolution would be extremely beneficial, enhancing our understanding of both fields and mechanistically explaining phenomena that depend on interactions between them. For example, a single model could explain both the evolutionary history and the commonness or rarity of species.

The study of ‘eco-evolutionary dynamics’ tackles the interaction between evolution and ecology over timescales of a few generations. For example, Thuiller *et al*. (2013) build local adaptation into metapopulation theory. However, macroevolutionary processes, such as speciation and extinction, act over millions of years, and are less well integrated with ecology (but see Vellend 2010). The study of community phylogenetics approaches this by adding phylogenetic information to community ecology (Webb *et al*. 2002; Cavender-Bares *et al*. 2009), but does not predict realistic macroevolutionary patterns as emergent behaviour over long time scales. Here, we construct such a model from first principles and use it to reveal new, testable predictions that span both ecology and macroevolution.

It is challenging to produce a theoretical basis for linking ecology and macroevolution because most pre-existing macroevolutionary theory is lineage-based and at the level of species or higher taxa, whereas ecology often focuses on individual organisms and their interactions. Individual-based macroevolutionary models are rarely studied because of their complexity although they are closer to reality and can include the individual-level processes that influence speciation and extinction. For example, more common species may be less likely to become extinct. A promising approach to develop a mechanistic model spanning ecology and macroevolution will therefore be to concentrate on the more fundamental individual organism level of community ecology and study the emergent macroevolutionary behaviour.

Neutral theory in ecology (Caswell 1976; Bell 2001; Hubbell 2001) yields a potential candidate for such a model in the form of the Unified Neutral Theory of Biodiversity and biogeography (UNTB) (Hubbell 2001), an individual-based model built around dispersal, speciation and demographical stochasticity (ecological drift). UNTB assumes that an individual organism's properties are independent of its species identity but still makes a rich variety of ecological and evolutionary predictions including species abundance distributions, beta diversity and species area relationships (Rosindell *et al*. 2011). Many macroevolutionary models, such as the birth-death model of diversification (Nee 2006) also assume neutrality, but at the species level.

On short timescales, neutrality may be a reasonable approximation: net fitness, averaged over time and space, cannot be very different between species without threatening coexistence. However, UNTB implicitly assumes something much less reasonable: individuals are neutral not only relative to their contemporaries, but also relative to all other individuals that ever lived. This relates to a recurring critique of UNTB: ecological drift without selection is too slow to explain deep time predictions (Lande *et al*. 2003; Nee 2005; Ricklefs 2006). In particular, species can only become common by following a sluggish random walk, which is inconsistent with the quick rise to dominance of certain species seen empirically (Leigh *et al*. 1993) and their observed declines to extinction in the fossil record (Lande *et al*. 2003).

Previous work enhanced UNTB by adding extra components, often producing models on a continuum with neutrality at one end; the challenge lies in choosing which additional factor to include. For example one can include niche structure (Purves & Pacala 2005; Gravel *et al*. 2006; Chisholm & Pacala 2010; Haegeman & Etienne 2011), direct fitness differences (Hubbell 2001; Zhou & Zhang 2008; O'Dwyer & Chisholm 2014), competitive asymmetries (Du *et al*. 2011; Haegeman & Loreau 2011; He *et al*. 2012; Pigolotti & Cencini 2013; Noble & Fagan 2015) or intraspecific density-dependence (Volkov *et al*. 2005; Du *et al*. 2011; Jabot & Chave 2011). Most work on neutral theory avoids phylogenetic predictions, even when longer timescales are being considered (Rosindell *et al*. 2010; O'Dwyer & Chisholm 2014). The few exceptions focused more on tree balance (Mooers *et al*. 2007; Jabot & Chave 2009; Davies *et al*. 2011) which is distinct from the timescale-related problems, for which the tree's branch lengths are central and where neutral theory performs poorly (Davies *et al*. 2011). Other authors incorporated more complex speciation mechanisms into individual-based models rather than building on UNTB (De Aguiar *et al*. 2009; Melián *et al*. 2012), but focused on the consequences of the speciation process for community ecology rather than phylogenies.

Here, we will construct a new individual-based Unified Theory of Ecology and Macroevolution (UTEM), by adding mild selection to UNTB. UTEM adds selection to UNTB in a very different manner to earlier work (Zhou & Zhang 2008; O'Dwyer & Chisholm 2014): in UTEM, fitness is hereditary and changes upon mutation. This enables us to reconcile approximately neutral ecological behaviour with faster temporal turnover of the community than would be expected under neutrality, arising from the build-up of selection over time. The individual-based nature of UTEM enables fitness to be considered at the level of individual organisms and so it can vary both within and between species. The addition of mild selection enables UTEM to produce more realistic macroevolutionary lineages-through-time (LTT) plots, and improved ecological predictions, especially species abundance distributions, compared to UNTB. We also find new emergent behaviours from the interaction between ecology and macroevolution that have broad implications for ecology and evolution. For example, larger communities tend to evolve fitter species compared to smaller communities.

## Methods

**Figure 1:**
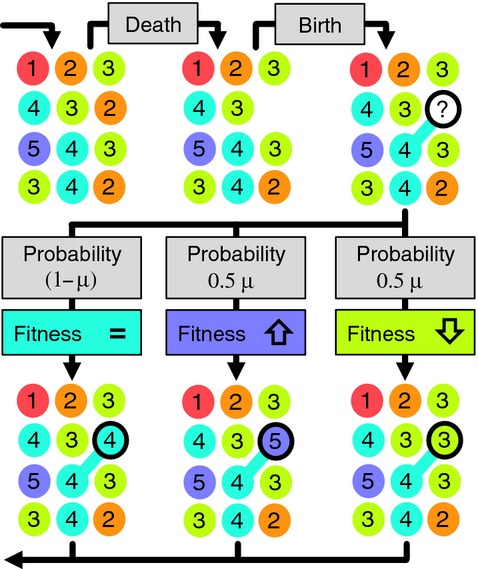
A description of one time step in our model for a simple example where metacommunity size *J*_M_ = 12. Each circle represents an individual organism. Species identities are not shown; the colours and numbers indicate the fitness category *c* for each individual. In this example, the dead individual had fitness category *c *=* *2 (orange) and is replaced with the offspring from an individual with *c *=* *4. The new-born individual inherits the fitness category of *c *=* *4; however, there is a small chance of a mutation to category *c *=* *5 or *c *=* *3.

In UTEM, there are four parameters: community size *J*_M_ ≥ 0, mutation rate 0 ≤ μ ≤ 1, selection strength *s *≥* *0, and a speciation threshold *n *>* *0. Each individual organism is assigned a ‘fitness weight’ indicating how well it competes against others; these are of the form (1 + *s*)^*c*^ where *c* is a variable ‘fitness category’ for each individual. Simulations are conducted in discrete time steps (Fig.1) each involving the random death of an individual, and replacement by the offspring of another. We focus on a non-spatial model to investigate predictions without the added complexity of space. An individual's probability of reproduction is thus proportional to its fitness weight but independent of its location. When *s *=* *0 UTEM reduces to a neutral model where all individuals compete equally. Like UNTB, our model assumes a constant *J*_M_, which can be considered as resulting from strong community-level density dependence.

Fitness category *c* is inherited by offspring, but with probability μ the offspring becomes a new ‘incipient species’ and either moves up or down by one fitness category with equal probability. The distribution of differences in *c* between individuals approaches an equilibrium state so the initial values of *c* are arbitrary, much like the initial species identities. Similarly, adding or subtracting a constant from the fitness category of all individuals makes no difference to the dynamics of the model because it changes all fitness weights by a fixed multiple leaving the actual probabilities of reproduction unchanged. Our simulations were run for a liberal burn-in period to reach their steady-state after which species abundances, phylogenies and individual finesses were periodically collected.

**Figure 2:**
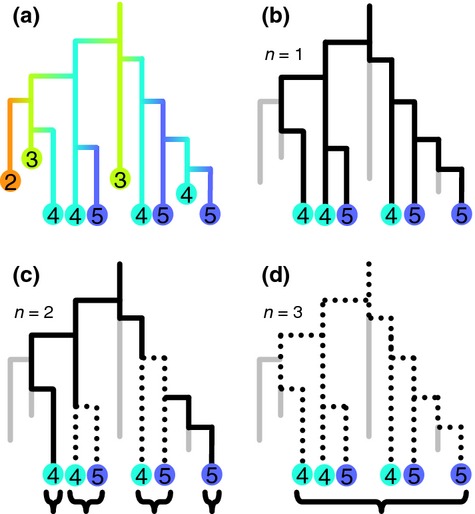
The definition of species in UTEM. Panel (a) shows an example simulation including all lineages, the numbers and colours on each shows the fitness category *c* of all its individuals. Horizontal links between lineages represent mutations causing a change in fitness category. In panels (b–d) we see the cases where *n *=* *1, 2 and 3, respectively, with extinct lineages removed. When *n *=* *1 we have six species. When *n *=* *2 we have four species; this can be checked by counting the number of horizontal moves in each path between tips of the tree. There are at least two mutations between good species, but only one between pairs of incipient species that connect the same good species. When *n *=* *3 all the incipient forms merge into one good species. The dotted lines indicate within-species structure of multiple incipient species, the black brackets indicate conspecific groups.

Individuals are considered to be of the same species in our model if there are fewer than *n* mutations along the genealogical path between them (Fig.2). Many different mutations could yield equivalent changes in fitness and so mutations are cumulative for defining species, even if they happen to return a lineage to its ancestral fitness category. In common with related work (De Aguiar *et al*. 2009; Melián *et al*. 2012), we resolve inconsistencies in the species definition by lumping uncertain groups into one species. Consequently, individuals with more than *n* mutations difference may be conspecific if other extant individuals bridge the gap between them. In the case where *n *=* *1, speciation reduces to UNTB's point mutation mode of speciation (Hubbell 2001). When *n *>* *1, however, UTEM instead replicates the properties of protracted speciation, where speciation takes time. In contrast to the original protracted speciation model with a fixed time to complete speciation (Rosindell *et al*. 2010), here time to complete speciation is a stochastic emergent quantity, similar to the case where incipient species pass through multiple stages (Etienne & Rosindell 2012). Evolution can occur within a single species when *n *>* *1 because new mutated incipient species may arise and others go extinct without them ever becoming sufficiently disconnected to be considered separate good species. As *n *→ ∞ there is only one species within which evolution of individual fitness continues, equating UTEM with familiar models in population genetics (Fisher 1930; Wright 1931; Ohta 1973; Desai *et al*. 2007).

Our model, is based on asexual reproduction to aid tractability, but can be considered as implicitly capturing properties of sexual reproduction such as the build-up of mutations leading to reproductive isolation and thus speciation. We are studying a community-level model so our ‘mutations’ should (especially when *n* and μ are not large) be interpreted as the appearance of a new incipient species, a much more significant change than the mutation of a single gene. For phylogeny construction we consider the time of divergence as the time of the first mutation (out of possibly many) that appears only in the species of interest and, if paraphyletic, any species nested within it. See Appendix S1 for detailed methods.

## Results

**Figure 3:**
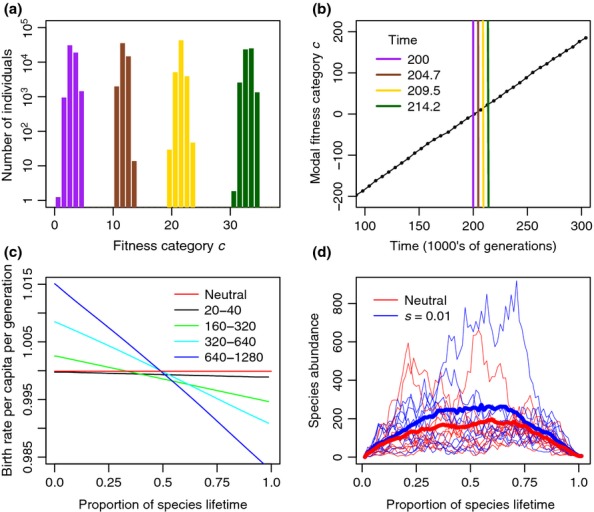
Fitness dynamics for parameters *J*_M_ = 100 000, μ = 0.0001, *n *=* *1, *s *=* *0.01. Panel (a) shows the number of individuals in each fitness category with colours corresponding to different times as indicated in panel (b). The black line in panel (b) shows the modal fitness category as a function of time for a single simulation. Panel (c) shows the mean net *per capita* birth rate for species over their lifespan from speciation (0) to extinction (1). This depends on the age that the species eventually attains (shown with different colours), the neutral case is included for comparison. Panel (d) shows how the abundance of a species varies over its life for species with lifespans in the range 640–1280 generations. The UTEM with *s *=* *0.01 (blue) is compared to the classic neutral model (red); thicker lines show average behaviour, whereas thin lines show individual species trajectories.

**Figure 4:**
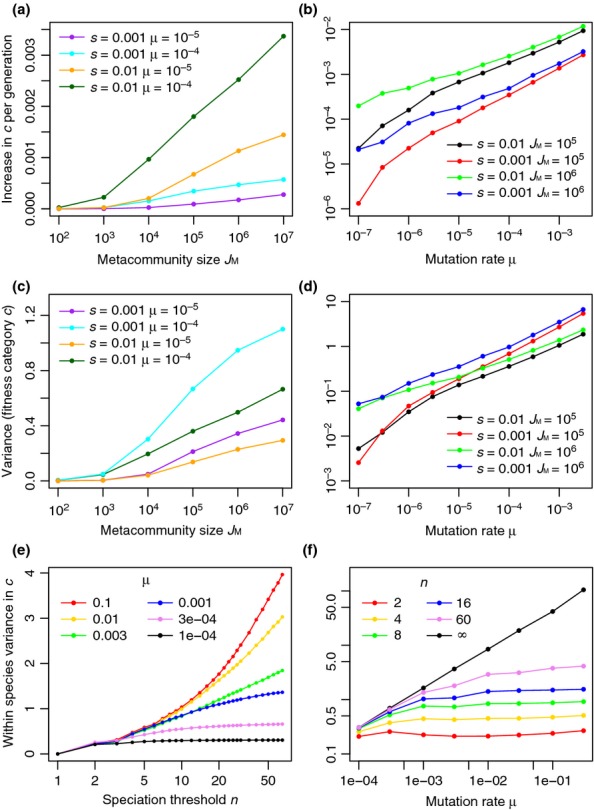
The effect of parameters *J*_M_, μ, *s* and *n* on the pace of evolutionary change (panels a, b), the variance in fitness category within the system (panels c, d) and the variance in fitness category within species (panels e, f). Panels (a, c) show the effect of metacommunity size *J*_M_ for a selection of *s* and μ values shown in different colours. Panels (b, d) show the effect of mutation rate μ for a selection of *s* and *J*_M_ values. In the case where *n *=* *1, these results refer to the fitness categories of good species; when *n *>* *1, they refer to the fitness categories of incipient species. Panels (e, f) show within-species variation in *c* for *J*_M_ = 10 000 and *s *=* *0.001 with variable μ and *n*. As *n →* *∞* all incipient species become lumped into one good species making the within-species variation equal to the variation in the entire system.

Simulations of our model show a tight distribution of incipient species fitness (Fig.3a) with a standard deviation that rarely exceeds one fitness category (Fig.4c, d). Individuals thus have similar fitness compared to others alive simultaneously in the same community. As time passes, however, the distribution of fitness categories moves (Fig.3a, b), allowing species to be very different to those alive a long time ago, whereas the shape of the fitness distribution remains relatively constant. The rate at which the modal fitness category progresses is not subject to much variation, even though the model is stochastic (Fig.3b).

When *n *=* *1 or when considering incipient species, the *per capita* birth rate for species that survive tends to start high and decrease steadily. The longest-lived species typically begin with a greater fitness advantage and thus a higher *per capita* birth rate (Fig.3c). The short-lived species often have a disadvantage from the start, which further deteriorates over time. In contrast, the *per capita* birth rate in a neutral model is independent of species age and identity (Fig.3c). On average, species of a given lifespan will reach higher abundances in UTEM than predicted under neutrality (Fig.3d). Species still follow a random walk in abundance, albeit a biased one inclined to increase for younger species and decrease for older species (Fig.3d).

The variance in the distribution of fitness categories is never large, but increases with increasing metacommunity size. The magnitude of this increase diminishes for larger metacommunities (Fig.4c). Variance in fitness also increases with increasing mutation rate, regardless of the other parameters. At small mutation rates the variance depends mostly on metacommunity size; at large mutation rates it depends mostly on the degree of selection *s*; there is a smooth transition between these cases at intermediate mutation rates (Fig.4d). Within-species variation in fitness category *c* is less than the variation across the entire system but the two converge as *n* increases and as μ decreases (Fig4e, f).

Individual organism fitness categories form a peaked distribution that moves in the direction of increasing fitness category forming a ‘travelling wave’ through time (Fig.3). The set speed of these waves indicates the speed of evolutionary change, which always increases with increasing metacommunity size regardless of the other parameters (Fig.4a). Larger mutation rates and stronger selection also increase the pace of evolutionary change. The relationship with metacommunity size becomes weaker for larger metacommunities (Fig.4a). For the largest mutation rates the speed depends mostly on selection strength, whereas for the smallest mutation rates it also depends on metacommunity size.

**Figure 5:**
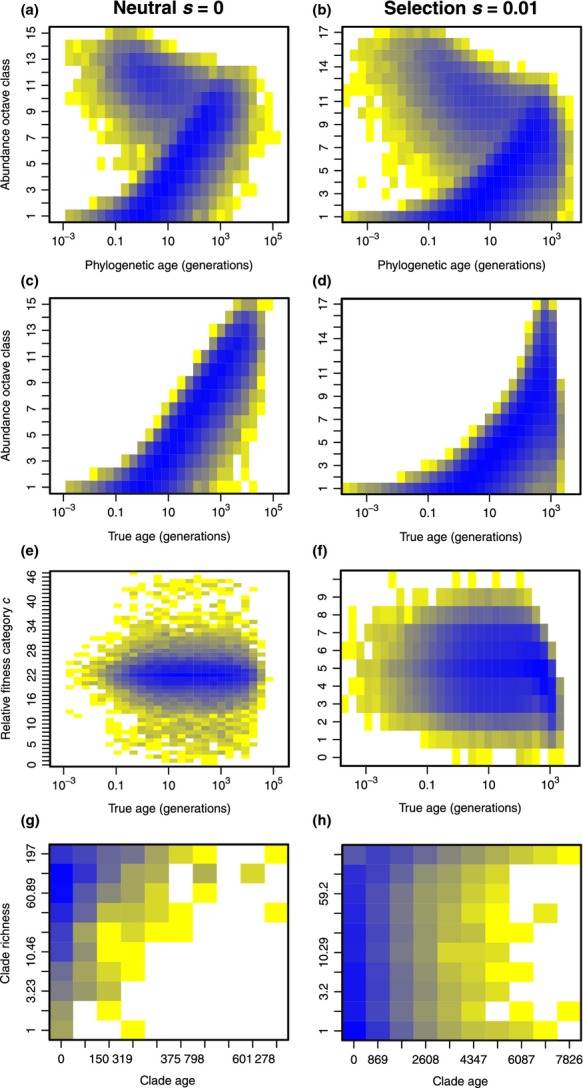
The relationship between age, fitness and abundance for species in a neutral model (panels a, c, e and g) and in UTEM with *s *=* *0.01 (panels b, d, f and h). Model parameters were *J*_M_ = 100 000, μ = 0.0002 and *n *=* *1. White corresponds to no species, whereas colours fading through yellow to dark blue correspond to increasing numbers of species on a logarithmic scale. Panels (a, b) show species abundance octave classes against phylogenetic age. Species with abundance between 2^(*i*−1)^ and 2^*i*^ − 1 fall in the *i*th abundance class. Panels (c, d) show octave class against true age. Panels (e, f) show fitness category against true age. Recall that in the neutral case where *s *=* *0 the ‘fitness category’ still exists and drifts at random without influencing the fitness weight. Panels (g, h) show the relationship between clade age and clade richness for every possible subclade of the complete phylogeny.

The way in which species ages and abundances are correlated depends on how ‘age’ is defined. Phylogenetic age is the time to the most recent divergence event from a *living* sister species or clade. The true age of a species is the time to the ‘incipient speciation’ event that first brought it into existence. The two can differ where a species arose from a now extinct sister species or has spawned extant sister species since its own origin. There is generally a humped relationship between abundance and phylogenetic age and a positive correlation between abundance and true age, however, selection induces a stronger non-linear relationship between true age on abundance (Fig.5a–d). In the neutral case when *s *=* *0, differences in fitness category do not translate to a change in fitness weight, so there is no relationship between fitness category and age (Fig.5e). When selection is introduced (*s *=* *0.01), however, we observe a decrease in fitness category for old species (Fig.5f) consistent with Fig.3c. In UNTB old clades tend to be species-rich compared to young clades, considering all possible subclades of the simulated phylogeny (Fig.5g). In contrast, the relationship between clade age and clade richness appears to break down under UTEM (Fig5h) consistent with recent findings for Eukaryotes (Rabosky *et al*. 2012) and with the existence of both living fossils and rapid radiations in the tree of life.

**Figure 6:**
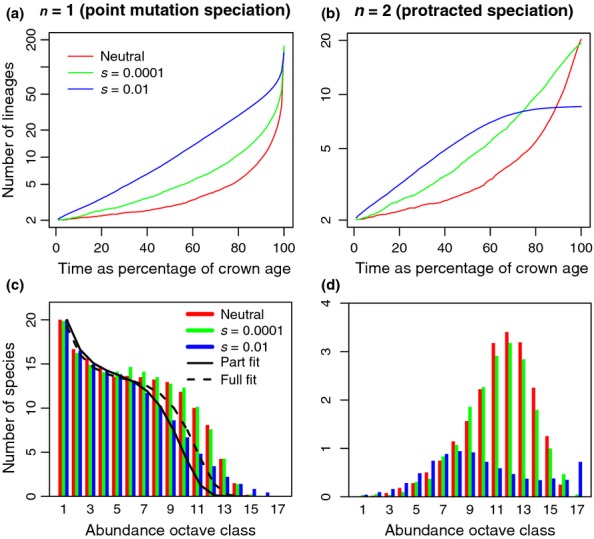
Evolutionary Lineages-Through-Time (LTT) plots (panels a, b) with ecological Species Abundance Distributions (SADs) (panels c, d). Model parameters were *J*_M_ = 100 000 and μ = 0.0002. Shown are the cases *n *=* *1 (panels a, c) and *n *=* *2 (panels b, d). The colours correspond to different degrees of selection and include the neutral case as shown in the legend. The log-series fit shown by the black line in panel (c) was the optimal least-squares fit considering only the first eight octaves of the SAD and not attempting to fit the remainder, which correspond to the most common species. The black dotted line shows an equivalent fit to the complete SAD. The blue bars in panel (c) show the case *s *=* *0.01, *n *=* *1 the first eight octaves of which was best fitted with a log-series where *J*_M_ = 11 622 and μ = 0.00172.

The introduction of selection in the UTEM has a profound impact on the relationship between time and number of extant lineages in a phylogeny: a Lineages Through Time (LTT) plot. The LTT plot from a neutral model (*s *=* *0) shows an extreme acceleration in diversification near the present day that is rarely seen empirically. As selection increases, the resulting LTT plots straighten and become akin to the those predicted by the quite different lineage-level birth-death model of diversification (Nee *et al*. 1994) (Fig.6a). The ecological predictions also change with increased selection, but not dramatically. The resulting species abundance distribution at equilibrium with *s *=* *0.01 is still log-series-like, but has an additional tail of really common species (Fig.6c), as is observed in reality but not predicted by UNTB (Ricklefs 2006; Etienne *et al*. 2007).

The acceleration in diversification near the present day on LTT plots remains when *n *=* *1 even if selection is relatively strong *s *=* *0.01. This phenomenon, known as the pull-of-the-present (Nee *et al*. 1994), also occurs in the birth-death model of diversification, and is caused by recently speciated lineages that have not yet gone extinct. When *n *=* *2 (protracted speciation), the pull-of-the-present disappears, but still only if there is selection as well (Fig.6c). The species abundance distributions for *n *=* *2 are different from a log-series and look qualitatively similar to a skewed lognormal distribution; however, they are more likely to be related to the ‘difference log-series’ (Rosindell *et al*. 2010) that emerges from UNTB with protracted speciation. The effect of increasing selection on this species abundance distribution increases the number of common species in this case creating a second mode in the distribution corresponding to the possibility of one species occasionally filling the entire community (Fig.6d).

## Discussion

Our model adds selection and a gradual ‘protracted speciation’ process to UNTB: small changes that yielded a substantial transformation in the model's behaviour. This shows that weak selection can enable individuals to have approximately equal net fitness over short timescales in combination with large differences over longer timescales. The gradual nature of speciation is taken into account by considering incipient species, which make up good species when lumped together in closely related groups.

New incipient species will on average have the same fitness as their parents because fitness is equally likely to increase or decrease. The addition of selection, however, favours those that happen to be fitter and so those young incipient species that survive extinction tend to have an advantage over older species (Figs3c and 5c, f). This advantage is small enough to retain a large portion of demographic stochasticity (Fig.3d), however, larger scale aggregate properties are noticeably affected.

The abundance of species tends to increase more at the start of their lives and decrease at the end; this is noticeable even in the neutral case, and amplified when selection is added due to the difference in *per capita* fitness between young and old species. These findings are consistent with those of Pigot *et al*. (2012) who showed that even stochastic models of range size evolution over a species’ life give rise to an apparently deterministic rise and fall when aggregated.

The distribution of different individual fitness categories in the entire community forms a peaked distribution (Fig.3a) with variation greater than that within individual species. Increasing the mutation rate increases the overall variation in fitness, but has less influence on within-species variation because species remain defined based on the accumulation of the same number of mutations (Fig4e, f). A fascinating emergent behaviour is that this distribution of fitnesses increases steadily, forming a travelling wave in fitness category through time. This is because incipient species with increased fitness are more likely to survive and pass their fitness on to descendants. The speed of travel increases with increasing mutation rate and metacommunity size (Fig.4) because of increased numbers of beneficial mutations.

Some of our results will be familiar to population geneticists, as it is known that population size affects the genetic evolutionary rate (Fisher 1930; Wright 1931; Ohta 1973) and in that similar travelling waves in fitness can emerge (Rouzine *et al*. 2003; Desai *et al*. 2007). The novelty of our work is in the suggestion that such results will apply at the very different scales of species (rather than genes) and communities (rather than populations), and in the addition of a speciation process to translate results into a macroevolutionary context. We expect that future developments building on our work will, inspired by the related results in population genetics (Rouzine *et al*. 2003; Desai *et al*. 2007), derive analytical approaches to evaluating UTEMs.

Our goal was to link our individual-based model of community ecology to macroevolution showing realistic behaviour naturally arising at macroevolutionary scales as an emergent result of simple processes. The LTT plots produced by our model are dramatically improved by the introduction of selection (Fig.6). UNTB predicts a faster than exponential rate of increase in the number of reconstructed lineages as a function of time, which is not generally observed (Phillimore & Price 2008). By setting *s *=* *0.01 we can recover LTT plots reminiscent of those from the lineage birth-death model of diversification (Nee *et al*. 1994) which has remained the standard phylogenetic model for decades. Selection with strength *s* = 0.01 for a system with 100 000 individuals is large by population genetics standards. However, in our ecological context a difference in fitness of 1% (*s* = 0.01) between individual organisms is likely to be challenging to detect from classic observations such as abundances, so our model can be considered *ecologically* ‘nearly neutral’.

In the case where *n* = 1, UTEM replicates the behaviour of the birth-death model of diversification, however, this includes the ‘pull-of-the-present’ (Nee *et al*. 1994), which is not typically seen in reality (Phillimore & Price 2008). The introduction of protracted speciation (*n *>* *1) is well suited to counteract this (Fig.6) because the pull-of-the-present is only observed near the present day where protracted speciation has the greatest effect by redefining recently split ‘species’ as ‘incipient species’. If we look further back in time than the time needed to complete speciation, every speciation process will have completed or led to extinction leaving little scope for protracted speciation to change the LTT plot. Neutral theory consistently produces misshapen LTT plots right from the root of the tree, which is too long ago to be influenced by protracted speciation. Consequently, both selection (*s *>* *0) and protracted speciation (*n *>* *1) are needed to obtain LTT plots that show a ‘slowdown’ in diversification.

Diversity-dependence (Valentine 1973) explains slowdowns in diversification by niche saturation. Another alternative explanation is changing parameters as a function of time. For example our preliminary work (Rosindell 2011) showed that constantly increasing metacommunity sizes in a neutral model can have a similar effect to that we have seen here by adding selection. However, UTEM can consistently produce phylogenies that display an apparent slowdown in diversification without requiring any temporally changing or non-equilibrium properties. More substantial evidence than a slowdown would thus be required to support less parsimonious non-equilibrium hypotheses.

The ecological predictions of UTEM are similar to the log-series predictions from UNTB at the metacommunity scale (Hubbell 2001); however, UTEM predicts more common species (Fig.6c). This is an improvement over UNTB, which performs badly when predicting the frequency of common species (Etienne *et al*. 2007). Ricklefs (2006) criticised UNTB because any abundant species would be unreasonably old and almost invincible to extinction. In UTEM, however, a species can become abundant by having a selective advantage and then decline quickly later in life from a selective disadvantage. Figure5 shows that older species are more abundant, but marginally less fit. This produces a signature of intraspecific density-dependence where more abundant species have reduced fitness; except that abundance does not directly cause reduced fitness, rather it implies age (because it still takes time to become abundant), and age in turn implies reduced fitness. O'Dwyer & Chisholm (2014) recently studied a model somewhat like ours, in which any new species was a marginally better competitor than all other species. They report slight differences in the ecological predictions compared to the original neutral theory, but their model shows fewer common species than UNTB, opposite to our observations. We attribute this difference to the fact that fitness is not hereditary in O'Dwyer and Chisholm's model; it is hereditary fitness that enables the accumulation of fitness advantages necessary to explain really common species.

Our model predicts that larger communities yield the evolution of species that, on average, are fitter compared to those from smaller communities. Consequently, in a connected system containing communities of different sizes, we speculate that diversity will naturally flow from the large communities to replace the inferior species in small communities. We introduce the terms ‘*source community*’ and ‘*sink community*’ to capture this. Once settled in a sink community, a lineage may diversify, but it is more likely to be outcompeted by a new lineage from a source community than it is to successfully emigrate. Diversity in a sink community is thus maintained by immigration and *in situ* speciation against heightened extinction rates. In contrast, source communities provide diversity to local sinks, have lower immigration rates and rely more on *in situ* speciation to maintain diversity. This suggests a tendency for surviving lineages to move from large areas to small areas rather than vice versa and may provide an explanation for the origin and destination of invasive species. It also has implications for native species in small isolated communities such as islands, which are likely to be community sinks: such species are expected to ultimately be outcompeted by superior mainland counterparts without human intervention.

The slowly progressing waves in fitness category over time in UTEM can be linked to Van Valen's (1973) Red Queen Hypothesis, encompassing the idea that species have to keep evolving to retain the same relative fitness. The term was motivated by a Lewis Carroll's Red Queen from ‘Alice through the looking glass’: **‘**Now, here, you see, it takes all the running you can do, to keep in the same place.’ The same concept emerges from UTEM: lineages need to keep up with the travelling wave to maintain the same relative fitness. The next words of the Red Queen are less well known but equally germane: **‘**If you want to get somewhere else, you must run at least twice as fast as that’. This encompasses a new feature of UTEM: to successfully invade another community, species typically need to have superior fitness and thus have come from a source community where the travelling wave moves faster.

Our findings are also in accord with those of Quental & Marshall (2013), who showed that the Red Queen Hypothesis can explain deterministic cycles of rise and fall of clade diversity observed in terrestrial mammals. Whilst the authors describe this as non-equilibrium behaviour, they are referring to diversity limits for clades that take some time to reach, after which the clade declines. In UTEM, species and clades can rise and fall as a result of Red Queen effects, but still within the context of a dynamic equilibrium and a fixed carrying capacity of individual organisms.

### Potential for testing and expanding UTEM

UTEM could be tested at three levels in future work: predictive, mechanistic, and conceptual. A predictive test requires choosing parameters that enable the model to reproduce empirical data. The parameters of our model may prove difficult to estimate other than through fitting. The same was true of the per capita speciation rate 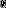 and metacommunity size *J*_M_ in UNTB; however, these parameters were ultimately only relevant as part of the fundamental biodiversity number 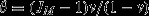 We expect that similar scaling properties will reduce the effective number of parameters in UTEM, enabling more effective fitting to empirical data and better parameter interpretation. For example if one decreases *s* and increases *n* and μ in the correct proportions, ‘mutations’ become more frequent but have less effect on fitness and speciation. We then expect that the discrete fitness categories would become more finely defined and ultimately continuous but nevertheless producing similar predictions. It may also be possible to estimate parameters by comparing patterns of genetic variation across and within species and through advances in understanding of the genomics of adaptation and speciation.

One way to test the mechanisms of our model would involve drawing competitive comparisons between individuals from different periods in evolutionary time. This cannot typically be done in the field (but see Rode *et al*. 2011); however, when conducting lab-based experiments with bacteria, it is possible to literally freeze some communities, whereas others can continue to evolve. Such experiments may support or refute the mechanisms behind our model and possibly show directly the predicted travelling waves of fitness. Indeed, existing work of this kind appears to support that absolute fitness continues to increase (Lenski *et al*. 1998). Furthermore, a population genetics model with similar properties to ours was verified by yeast population experiments (Desai *et al*. 2007). Outside of the microbial world, we expect that increasing quantities of empirical data from animals and plants will lead to a better understand about adaptation and selection and thus have the potential to confirm the mechanisms behind UTEM.

Finally, the finding that species fitness increases slowly at speeds depending on community size and that this will lead to the emergence of ‘community sources’ and ‘community sinks’ transcends the precise model details. A fundamental question in ecology and evolution is to determine the movement of species over space and time. Such analyses are challenging, but progress continues to be made by use of a combination of phylogenetic, range size and paleontological data (Price *et al*. 2014). The patterns unveiled by such analysis will aid in understanding the conceptual essence of UTEM and our predictions of community sources and sinks after the confounding effects of community size (such as differing numbers of potential dispersers) are properly accounted for.

Future enhancements to UTEM could consider the effects of spatial structure, enabling our predictions regarding the geographical movement of lineages to be verified. An investigation into how the predictions of UTEM change with system size and increasing *n* is also interesting. Our simulations of systems with 100 000 individuals are rather small and consequently have fast overall dynamics even though these dynamics are correct in proportion to one another. We expect larger systems will produce similar behaviour, but with overall temporal turnover scaled back. Revisiting UTEM from the context of sexually reproducing organisms, and including asymmetric effects of mutation on fitness also seem interesting research directions, although this would come at the cost of increasing complexity and may not be so relevant at the broad scales considered here.

Finally, multiple environmental types could be included with each individual having a different fitness category for each environment. Studying the effect of environmental stochasticity is also a promising research direction. Our preliminary work showed that changing community size on its own is unlikely to produce satisfactory results, but environmental stochasticity in multiple environmental types, where each individual has a fitness weight that depends on the environment it is in, may be more productive. Such analyses would enable us to see how temporally and spatially varying environmental conditions might affect whether communities classify as sources or sinks of species.

## Conclusion

We have made a first attempt at a new class of model: an individual-based model of ecology and macroevolution (UTEM) built from the foundations given by UNTB. Our model behaves similarly to UNTB over ecological time scales but differently over evolutionary timescales, and provides improved understanding of why UNTB produces realistic predictions whilst being based on fundamentally false assumptions. UTEM resolves several problems with UNTB relating to the distortion of timescales, and at the same time makes new and far-reaching predictions. In particular, we recover LTT plots consistent with the birth-death model of diversification, but with the potential to display a slowdown in diversification. Our model also explains the presence and demise of abundant species in reasonable timescales. Perhaps most interestingly an emergent behaviour is a wave of evolutionary change at the community level that travels at a speed dependent on community size. This result is analogous to that found in population genetics, but applies here at the very different scales of community ecology and macroevolution and provides a simple explanation for the ‘Red Queen’ models of macroevolutionary dynamics. We suggest that the concepts of a slowly moving wave of evolutionary change at the community level, leading to the idea of community sources and sinks will lead to a new perspective on a range of biological problems continuing the legacy of UNTB. Ultimately UTEM may have a role in explaining phenomena such as the origin and spread of invasive species, and the historical movement of lineages through space in the fossil record.
