# Supplementary material for: Unifying ecology and macroevolution with individual-based theory
Source: Ecol Lett. 2015 Mar 27;18(5):472–82. doi: 10.1111/ele.12430 (PMC4403962; doi:10.1111/ele.12430)
Supplement: Supplementary file 1 [file ele0018-0472-sd1.docx]

**Appendix A: Full description of methods**

***Parameters***

Our simulations are conducted based on four input parameters:

**Community size *J*_M_ > 0**: the total number of individual organisms in the community. This number does not vary during the simulation for the present work, but could be allowed to do so in future extensions.

**Mutation rate** $\boldsymbol{0\leq}\boldsymbol{\mu}\boldsymbol{\leq1}$: the rate at which individual lineages accrue mutations. These mutations cause changes in fitness and accumulate to lead to speciation.

**Selection strength** $\boldsymbol{s\geq0}$: each individual organism is assigned a ‘fitness weight’ indicating how well it will compete against others; these are of the form ${(1+s)}^{c}$ where *c* is a variable ‘fitness category’, different for each individual. When$s=0$ this reduces to a neutral model in which all individuals compete equally regardless of their values of *c*.

**Speciation threshold** $\boldsymbol{n>0}$: the minimum number of mutations between any pair of individuals of different species. Any pair of individuals must be conspecific if there are fewer than *n* mutations between them. If there are more mutations between them they may still be conspecific depending on whether or not it’s possible to split them into two species without breaking the rule described above.

***Dynamics***

All our simulations have an initial condition. This consists of species identities (arbitrary labels) and values of *c* (fitness categories) for every one of the *J_M_* individuals in the system. The effect of the initial condition decays with time as the system reaches equilibrium just as was the case with the unified neutral theory of biodiversity and biogeography. We chose a mono-dominant initial condition with all individuals having the same value of *c* and the same species identity. Simulations from which we collected phylogenetic data were run for a liberal burn-in period: quadruple of that required for the descendants of one individual alive in the initial condition to fill the landscape. Simulations from which we only collected information on the distribution of fitness categories *c* were run for a slightly less stringent burn in period of at least 2*J_M_* ^2^ time steps. This was because we found that these results reached equilibrium much faster than those involving phylogenies.

All simulations are conducted forwards in discrete time steps. In each step an individual dies, chosen according to a uniform distribution from the metacommunity containing *J*_M_ individuals. This leaves a gap that will be filled immediately by the offspring of another individual. An individual’s probability of being the one to place its offspring in the gap is equal to its fitness weight divided by the sum of the fitness weights of all other individuals (excluding the one that just died). Adding or subtracting a constant from the fitness category of all individuals will thus have no influence on the overall dynamics: it will result in all fitness weights changing by a fixed multiple only and thus the probabilities of reproduction after normalisation will remain the same. We focus on a non-spatial model to investigate the predictions of UTEM without the complexity of space. This means that each individual’s probabilities of death and reproduction were independent of its position in space, we could relax this assumption in future work.

Any newly born individual will inherit the fitness weight of its parent with probability $(1-\mu)$. Otherwise, with probability $\mu$ it will mutate and acquire a new fitness weight: if the parent’s fitness weight was ${(1+s)}^{c}$ then the mutated offspring’s fitness weight is equally likely to be either ${(1+s)}^{c+1}$ or ${(1+s)}^{c-1}$. Many different mutations could yield changes in fitness and so a set of mutations are still considered to have led to change in a lineage even if its fitness category happens to have returned to an ancestral value.

***Species definition***

Species identity in our model is defined by $n$: individuals are considered to be of the same species if there are fewer than *n* mutations along the genealogical path between them. In common with other metacommunity models incorporating speciation, we resolve inconsistencies in the species definition by lumping uncertain groups into single species (de Aguiar *et al.* 2009; Melián *et al.* 2012). Consequently, individuals with more than *n* mutations between them may be conspecific where other extant individuals bridge the gap between them with links each containing sufficiently few mutations.

In the special case where $n=1$, our model reduces to UNTB’s point mutation mode of speciation (Hubbell 2001). When $n>1$, however, our model instead replicates the properties of protracted speciation, where the speciation process takes time to complete. In contrast to the original protracted speciation model where the time to complete speciation was fixed (Rosindell *et al.* 2010), here it is a stochastic emergent quantity, similar to the case where incipient species pass through multiple stages en route to separation (Etienne & Rosindell 2012).

***Mutation definition***

Although we use the term ‘mutation’, we are studying a community-level model and this refers to a more significant change than the mutation of a single gene, especially where *n* and $\mu$ are large. Our mutations correspond instead to the arrival of a new incipient species. Evolution can occur within a single species when *n* > 1 because incipient species can arise and go extinct enabling change within the species without becoming sufficiently disconnected to be classified as good species. Indeed, as $n\to\infty$ every individual becomes classified as a single species within which evolutionary change still continues.

***Phylogeny definition***

To define a dated phylogeny, we require a date for each species, which defines when it split from its sister clade. The start date for a good species is less well defined when $n \geq2$ because species may split simply by the extinction of a connecting incipient species (Melián *et al.* 2012). Each good species will have a number of mutations that appear only in that species (and any paraphyletic species nested within it). We took the earliest date of any of these mutations as the date of origin of the species for the purposes of phylogeny construction. Time was measured in generations where each time step was 2/*J*_M_ generations (note that generations were overlapping hence the factor of 2 here).

Our method makes paraphyletic species possible but not polyphyletic species, because at every node in the phylogeny (looking back from the present day) we either define a monophyletic group that has no other conspecifics or join two sister lineages to become conspecific. The date of common ancestry between a paraphyletic species and a good species nested within it will be the date of the earliest mutation that appears only in the nested species.

***Data collection and data processing***

After burn in, our main simulations periodically recorded the abundance and fitness category of every incipient species and produced a Newick format phylogenetic tree. All Individuals in the system were sampled and contributed to the data. These data were then post processed for different values of n at a later stage with incipient species being lumped appropriately. This approach was for technical convenience, it would have been possible though computationally more expensive, to track species identity continuously and independently for each value of *n*. The periodically collected data from the system was later averaged for producing graphs.

For patterns of clade richness against clade age we took every possible sub clade from every simulated phylogeny with the specified parameters and binned this in categories based on their richness and age.

We also run a simpler alternative form of simulation that only tracked the individual values of c and no other features - this enabled efficient production of figure 3 and part of figure 4 in the main text without unreasonable computational expense on the larger community sizes.

***Software and hardware details***

The main simulations were conducted using C++ and run on a high performance computing facility using approximately 3 days of CPU time on every set of parameters. We did not use coalescence-based algorithms because these are not suitable for performing our type of simulation in their classic form (Rosindell et al. 2008). As part of future work it may be possible to develop coalescence-based algorithms for these simulations. Post-processing of data and plotting of graphs were conducted using R (R Core Team 2012), some of which was also conducted using high performance computing and some of which was run locally. We used the libraries APE (Paradis E et al. 2004) and apTreeshape (Bortolussi et al. 2012) in R for basic phylogeny manipulation and Lineage Through Time (LTT) plot production.

***References***

1. Bortolussi, N., Durand, E., Blum, M. & Francois, O. (2012). apTreeshape: Analyses of Phylogenetic Treeshape. R package version 1.4-5.
2. De Aguiar, M.A.M., Baranger, M., Baptestini, E.M., Kaufman, L. & Bar-Yam, Y. (2009). Global patterns of speciation and diversity. *Nature*, 460, 384–7.
3. Etienne, R.S. & Rosindell, J. (2012). Prolonging the past counteracts the pull of the present: Protracted speciation can explain observed slowdowns in diversification. *Syst. Biol.*, 61, 204–213.
4. Melián, C.J., Alonso, D., Allesina, S., Condit, R.S. & Etienne, R.S. (2012). Does sex speed up evolutionary rate and increase biodiversity? *PLoS Comput. Biol.*, 8, e1002414.
5. Paradis E., Claude J. & Strimmer K. (2004). APE: analyses of phylogenetics and evolution in R language. Bioinformatics 20: 289-290.
6. R Core Team (2012). R: A language and environment for statistical computing. R Foundation for Statistical Computing, Vienna, Austria. ISBN 3-900051-07-0, URL.
7. Rosindell, J., Cornell, S.J., Hubbell, S.P. & Etienne, R.S. (2010). Protracted speciation revitalizes the neutral theory of biodiversity. *Ecol. Lett.* 13, 716-727.
8. Rosindell, J., Wong, Y. & Etienne, R.S. (2008). A coalescence approach to spatial neutral ecology. *Ecol. Inform.*, 3, 259–271.
